# Engagement in a virtual group-based walking intervention for persons with schizophrenia: a qualitative study

**DOI:** 10.1186/s12888-024-06250-9

**Published:** 2024-11-11

**Authors:** Julia Browne, Claudio Battaglini, Aslihan Imamoglu, Bryan Stiles, L. Fredrik Jarskog, Paschal Sheeran, Ana M. Abrantes, Tonya Elliott, Oscar Gonzalez, David L. Penn

**Affiliations:** 1 Center of Innovation on Transformative Health Systems Research to Improve Veteran Equity and Independence (THRIVE COIN), VA Providence Healthcare System, 830 Chalkstone Ave, Providence, RI USA; 2 Department of Psychiatry and Human Behavior, Alpert Medical School of Brown University, Providence, RI USA; 3 Department of Exercise and Sport Science, University of North Carolina at Chapel Hill, Chapel Hill, NC USA; 4 Department of Psychology and Neuroscience, University of North Carolina at Chapel Hill, Chapel Hill, NC USA; 5 Department of Psychiatry, University of North Carolina at Chapel Hill School of Medicine, Chapel Hill, NC USA; 6 School of Behavioural and Health Sciences, Australian Catholic University, Melbourne, VIC Australia

**Keywords:** Exercise, Participation, Attendance, Barriers, Facilitators, Rapid analysis

## Abstract

**Background:**

Exercise is beneficial for persons with schizophrenia; however, high dropout rates limit the impact of interventions. Virtual exercise programs have the potential to improve engagement; however, few intervention studies of virtual programs have been conducted in this population.

**Methods:**

This study examined qualitative data from 15 adults with schizophrenia who participated in a pilot randomized controlled trial of Virtual PACE-Life, a live, video-delivered group walking intervention guided by self-determination theory. Interviews elicited feedback on the intervention, barriers and facilitators to engagement, recommendations for intervention refinement, and preferences for exercise programming modality. Rapid qualitative analysis was used to explore similarities and differences between completers (i.e., those that attended *≥* 50% of virtual walking sessions; *n* = 9) and non-completers (i.e., those that attended < 50% of virtual walking sessions; *n* = 6).

**Results:**

Both groups viewed Virtual PACE-Life positively but found the virtual exercise sessions challenging and inadequate for facilitating social interaction. Work obligations impacted completers’ attendance whereas technological issues and forgetting impacted non-completers’ attendance at virtual walking sessions. Completers preferred virtual exercise programs and non-completers preferred in-person exercise programs.

**Conclusions:**

These findings suggest that future virtual group-based walking programs should prioritize enhancing the social aspect, offering scheduling choices, and regularly assessing the perceived difficulty of exercise sessions. These modifications not only have the potential to improve intervention engagement but they also may increase participant autonomy and relatedness, core components of self-determination theory.

## Background

Exercise has multidimensional benefits for adults with schizophrenia including improved cardiorespiratory fitness, mental health symptoms, functioning, quality of life, and cognition [1–3]. Exercise interventions are especially critical for this population given the high levels of sedentary time, low levels of physical activity, and excess medical burden observed in people with schizophrenia [4, 5]. Reduced engagement in health-promoting behaviors and high rates of chronic medical conditions (e.g., cardiovascular disease) contribute to premature mortality of those with schizophrenia [6–8]. Despite efforts to develop and test exercise programs for this population [9], the dropout rate remains elevated, thereby limiting the impact of existing interventions. In fact, a meta-analysis of 19 randomized controlled trials of physical activity programs for people with schizophrenia found a dropout rate of 27% [10], which is higher than those found in other populations with mental health conditions such as those with depression (18%) [11] and anxiety and stress-related disorders (22%) [12]. Therefore, identification of the factors contributing to the elevated dropout rates is needed to facilitate greater engagement in exercise programs for adults with schizophrenia.

Low mood, stress, reduced motivation, and lack of support make it difficult for people with schizophrenia to engage in exercise [13, 14]. Yet, at the same time, these aspects are among the prominent reasons that people with schizophrenia do exercise along with a desire to improve their physical health [13, 15, 16]. As such, social support from peers and supervision from intervention leaders have emerged as key ingredients for enhancing engagement in exercise programs designed for those with schizophrenia [17–19]. Though support can help address personal and social barriers to exercise, these may not be sufficient to overcome environmental obstacles affecting this group, such as limited income/resources and transportation difficulties [20, 21].

Delivering interventions remotely (e.g., through digital and/or telehealth platforms) is one strategy to enhance access and limit the need for transportation. During the COVID-19 pandemic, we converted an in-person group walking intervention for people with schizophrenia (Physical Activity Can Enhance Life [“PACE-Life”]) to a fully virtual program (“Virtual PACE-Life”). Virtual PACE-Life, a live, video-delivered group-based walking program for adults with schizophrenia was then tested in a pilot randomized controlled trial (RCT) compared to an activity tracking control condition (“Fitbit Alone”). Although results of the pilot RCT showed no differences in cardiorespiratory fitness or physical activity between groups, attendance in Virtual PACE-Life was higher (56%) [22] than in an open trial of the in-person version of PACE-Life (34%) [23]. Yet, it should be noted that Virtual PACE-Life was also shorter (16 weeks) than in-person PACE-Life (24 weeks), which could have affected these attendance rates. Analyses of outcomes by engagement showed greater benefits for Virtual PACE-Life completers (i.e., those that attended 50% or more virtual walking sessions) on physical activity and exercise motivation compared to non-completers (i.e., those that attended < 50% of virtual walking sessions) [22]. Therefore, identifying the factors that both facilitate and hinder engagement in Virtual PACE-Life would be valuable for future efforts devoted to disseminating remote exercise programs for the schizophrenia population.

The purpose of the present study was to explore participant qualitative feedback on the Virtual PACE-Life intervention. The study aims were to explore the similarities and differences in (a) views of the intervention components, (b) barriers and facilitators to engagement, (c) recommendations for intervention refinement, and (d) preferences for exercise programming modality between Virtual PACE-Life completers and non-completers.

## Methods

### Study design

The present study is an analysis of qualitative data from a 16-week two-arm parallel pilot RCT (Virtual PACE-Life vs. Fitbit Alone). The pilot RCT of Virtual PACE-Life versus Fitbit Alone took place in a single US state between January 2021 and March 2022 and was registered on clinicaltrials.gov (NCT04173572; initial registration on 11/27/2018). All research was perfomed in accordance with the Declaration of Helsinki and was approved by the University’s Institutional Review Board. Main outcome results from the pilot RCT have been published [22]. This qualitative analysis included only participants randomized to the Virtual PACE-Life condition.

### Participants

Eligibility criteria for the pilot RCT were: (1) schizophrenia-spectrum disorder diagnosis, (2) at least a 4th grade reading level, (3) clinically stable (i.e., no psychiatric hospitalizations in prior three months or psychiatric medication changes in prior month), (4) low engagement in moderate-intensity exercise (i.e., less than 60 min/week over prior six months), and (5) medically safe to participate in a walking program.

Out of 73 individuals screened for the RCT, 37 were eligible and were subsequently enrolled. These 37 participants were randomized to Virtual PACE-Life (*n* = 17) or Fitbit Alone (*n* = 20). Out of the 17 Virtual PACE-Life participants randomized, 15 completed the qualitative interview and thus were included in this analysis (Note: one participant withdrew from the study and one participant was unable to be reached for the 16-week assessment).

### Intervention

Virtual PACE-Life is a virtual group-based walking intervention for adults with schizophrenia guided by self-determination theory [24, 25], a theory of motivation (involving fulfillment of relatedness, autonomy, and competence needs), to improve cardiorespiratory fitness. Detailed information regarding the intervention components, leader training, and fidelity monitoring have been published elsewhere [22].

Virtual PACE-Life involves live, video-delivered group walking sessions held twice/week, recommendations and resources for home-based walking sessions, activity tracking with Fitbits, and motivational strategies (i.e., goal-setting, problem-solving, social support). Virtual walking sessions were conducted via Zoom by trained group leaders. All group leaders, who were mental health clinicians or graduate/undergraduate students (in clinical psychology or exercise and sport science), received initial training, attended weekly supervision calls during the study, and were monitored for fidelity. Zoom sessions included walking-based exercises (e.g., walking in place, side steps, kicks, knee raises) which initially lasted 15 min but slowly increased over the course of the 16 weeks to 30 min in duration. Participants were provided with recommendations for individualized heart rate zones [26] and rates of perceived exertion [27] to guide their walking session intensity, both of which increased gradually over the 16 weeks to promote an exercise dose-response. At least two group leaders conducted all walking sessions. On average, four participants (SD = 1.4, range: 0–7) attended each session.

In addition to virtual sessions, group leaders provided recommendations for the number, duration, and intensity of home-based walking sessions (which also gradually increased over the course of the study) and provided participants with a list of freely available walking-based exercise videos to use at home. To enhance motivation, participants were encouraged to set goals for the number of steps/day and the number of home-based walks they would complete over the next week. Goal-setting, which occurred in the virtual group setting, involved participants setting their goals, providing feedback to others on their goals, and reflecting on their progress from the prior week. Twice during the 16 weeks, participants independently identified barriers to walking and solutions to address the barriers in the form of if-then plans (i.e., “if [barrier] then [solution]”) [28]. If-then plans were developed through online surveys sent to participants and were not discussed during groups. Finally, social support from group leaders and among peers was emphasized during all group sessions. Specifically, group leaders provided verbal praise and encouragement during walking sessions (e.g., “excellent job, keep it up”) as well as during goal-setting sessions (e.g., “great work reaching your step goal this week”). Group leaders and participants provided behavioral displays of support (e.g., clapping) during sessions. Moreover, group leaders facilitated peer-to-peer support during goal-setting sessions by asking participants to comment on their fellow participants’ progress (e.g., “what do you think of how [participant] was able to reach their goal this week?”).

### Measures

The research team developed a semi-structured interview guide to ensure the consistency of qualitative interview administration. The interview guide included 21 questions aimed to elicit feedback on the following areas: (1) components of the Virtual PACE-Life Intervention (i.e., overall experience and specific feedback on virtual walking sessions, group leaders, peer social support, goal-setting sessions, Fitbit devices, and home-based walking sessions), (2) barriers and facilitators to engagement in Virtual PACE-Life (i.e., virtual and home-based walking sessions), (3) obtaining recommendations for refinement of Virtual PACE-Life, and (4) identifying preferences for exercise programming modality (i.e., in-person or virtual). The interview guide included both open-ended and close-ended questions with opportunities for follow-up questions to clarify responses and/or to obtain additional information (Table 1).

**Table 1:** Qualitative semi-structured interview guide

***Feedback on the Virtual PACE-Life Intervention***
1. How did you find the PACE-Life program? 2. If someone asked you to tell them about PACE-Life, what would you say? 3. What did you think of the virtual exercise sessions delivered on zoom? a. Follow-up questions: Was it worth doing? Enjoyable? Was there a good balance between exercise and enjoyment? 4. What did you think of the goal-setting sessions held after exercise? 5. How did you feel about the group leaders? a. Follow-up questions: How did the leaders make you feel about the group sessions? If you were in charge, is there anything you’d change about how the leaders run the session? 6. Did you feel you got to know the other group members? a. Follow-up question: Did you feel that you were part of a ‘group’? 7. What did you do for your independent exercise sessions (e.g., walk outside, use online videos)? a. Follow-up question: How did you decide what to do? 8. What was your experience like using a Fitbit? a. Follow-up question: Is there anything that should have been explained a bit more about using the Fitbit? 9. What did you like about the PACE-Life intervention? Why? a. Follow-up question: If you were asked to recommend PACE-Life to friend, what would you say? 10. What didn’t you like about the PACE-Life intervention? Why? 11. Did you notice any changes in your life while you were in the PACE-Life intervention? What were they? a. Follow-up question: why do you think this happened?
***Barriers and Facilitators to Engagement in Virtual PACE-Life***
12. How easy or difficult did you find it to attend the virtual exercise sessions? 13. If you missed any sessions, why did this happen? a. Follow-up question: Is there anything you could have done to avoid this happening? 14. How did you motivate yourself to the virtual exercise sessions, what did you tell yourself? 15. If there were times you didn’t feel like doing the virtual exercise sessions, how did you get yourself to do them? 16. How easy or difficult did you find it to attend the independent exercise sessions? 17. If you missed any sessions, why did this happen? a. Follow-up questions: Is there anything you could have done to avoid this happening? Did you try to make up the sessions later? 18. How did you motivate yourself to do the independent exercise sessions, what did you tell yourself? 19. If there were times you didn’t feel like doing the independent exercise sessions, how did you get yourself to do them?
***Recommendations for Intervention Refinement and Modality Preference***
20. What should we do to make PACE-Life better in the future? Why? 21. If you had the option to have group walks in person or virtually (on Zoom), which would you prefer? Why?

### Procedure

Eligible participants completed the baseline assessment in person or virtually depending on participant preference. Randomization (1:1 to Virtual PACE-Life or Fitbit Alone) was conducted at the end of the baseline assessment. Participants randomized to Virtual PACE-Life were provided with a technology introduction to using Zoom and the Fitbit device (Fitbit Charge version 3 or 4) as well as offered a tablet and data plan if needed (all conducted by unblinded research staff). Blinded research staff administered post-baseline assessments (at 8 weeks, 16 weeks, and 20 weeks) of physical health and psychosocial measures.

Qualitative interviews were part of the 16-week assessment for Virtual PACE-Life participants; however, to maintain research staff blinding for the 20-week assessment, an unblinded research staff member coordinated scheduling and administration of these interviews. Interviews were conducted virtually via Zoom within one month of the final scheduled virtual walking session. Participants had the option to have their camera on or off during interviews; however, only the audio recording of the interview was downloaded and subsequently manually transcribed. Interviews lasted, on average, 24.9 min (SD = 11.7).

### Data Analysis

Three members of the research team analyzed the interview transcripts using a rapid qualitative analysis approach. This approach includes summarizing responses to the interview questions (i.e., data reduction) using a structured form (i.e., templated summary) and then visualizing the responses in a grid (i.e., matrix display) [29–31].

Consistent with this approach, the lead author developed a templated summary form that included domains aligned to the interview questions for summarizing the transcripts (Table 2). All three research team members reviewed the templated summary, independently used it to analyze three transcripts, and then met together to discuss the templated summary form and the responses to the three transcripts. The research team determined that the templated summary form was adequate and developed specific guidelines for maintaining consistency of data reduction including using short phrases to summarize transcripts with no more than three summary phrases per domain. The remaining 12 transcripts were summarized by two team members using the templated summary. Every two weeks during the analysis phase, the three research team members met as a group to review the templated summaries (completed by at least two team members) and addressed any discrepancies observed between the two raters. The third rater (that did not summarize the transcript being discussed) served as the decider for any discrepancies. The templated summaries were transferred to an overall matrix display of each domain by each transcript. The lead author then divided the complete matrix display into two displays to allow for exploration of summaries for completers (i.e., participants that attended 50% or more virtual walking sessions; *n* = 9) and non-completers (i.e., participants that attended < 50% of virtual walking sessions; *n* = 6). The research team then met as a group to review the two matrix displays to identify key responses across domains for completers and non-completers.

**Table 2:** Templated Summary and Matrix Display domains and interview items

Domain	Aligned Interview Question(s) (from guide shown in Table 1)
***Feedback on the Virtual PACE-Life Intervention***	
Overall experience of Virtual PACE-Life	1, 2, 9, 10, 11
Feedback on virtual walking sessions	3
Feedback on goal-setting sessions	4
Feedback on groups leaders	5
Feedback on peer social support	6
Feedback on home-based walking sessions	7
Feedback on Fitbits	8
***Barriers and Facilitators to Engagement in Virtual PACE-Life***	
Barriers to virtual walking sessions	12, 13
Barriers to independent (home-based) walking sessions	16, 17
Facilitators to engagement in walking sessions	14, 15, 18, 19
***Recommendations for Intervention Refinement and Modality Preference***	
Recommended changes to Virtual PACE-Life	20
Exercise programming modality preference	21

## Results

### Participants

Completers (*n* = 9) attended an average of 77.7% (SD = 13.6, range: 50.0 – 90.7%) of virtual walking sessions. Participants in this group were, on average, 42 years old (SD = 10) and not Hispanic or Latinx (78%). With respect to gender and race, the sample was 56% female and 44% male, and 44% White and 44% Black or African American (11% Mixed). Just over half of the sample had a college degree or higher (55%) and 44% were employed. All completers were taking antipsychotic medications and all but one participant had a baseline body mass index (BMI) in the overweight or obese range (i.e., at or above 25.0) (Note: baseline BMI was missing for two participants).

Non-completers (*n* = 6) attended an average of 20.2% (SD = 19.7, range: 3.1 – 46.4%) of virtual walking sessions. Participants in this group were, on average, 41 years old (SD = 15) and not Hispanic or Latinx (83%). With respect to gender and race, the sample was 33% female and 67% male, and 50% White and 33% Black or African American (17% Mixed). 67% had some college education (33% had a college degree) and 83% were not employed (Table 3). All non-completers were taking antipsychotic medications and all had a baseline BMI in the overweight or obese range (i.e., at or above 25.0).

**Table 3:** Demographic characteristics of virtual PACE-Life completers and non-completers

Characteristic	Completers (*n* = 9)	Non-completers (*n* = 6)
Age (years), M (SD)	41.8 (10.3)	40.5 (14.7)
Gender, n (%)		
Female	5 (55.6)	2 (33.3)
Male	4 (44.4)	4 (66.7)
Race, n (%)		
White	4 (44.4)	3 (50.0)
Black or African American	4 (44.4)	2 (33.3)
Mixed	1 (11.1)	1 (16.7)
Ethnicity, n (%)		
Hispanic or Latinx	2 (22.2)	1 (16.7)
Not Hispanic or Latinx	7 (77.8)	5 (83.3)
Education, n (%)		
High school diploma or equivalent	2 (22.2)	0 (0.0)
Some college	2 (22.2)	4 (66.7)
College degree	3 (33.3)	2 (33.3)
Higher than college	2 (22.2)	0 (0.0)
Employment, n (%)		
Employed	4 (44.4)	1 (16.7)
Not Employed	5 (55.6)	5 (83.3)

Note Percentages may not add to 100 due to rounding

### Feedback on the virtual PACE-Life intervention

#### Completers

In terms of the specific components of PACE-Life, completers found the virtual walking sessions motivating, enjoyable, and helpful but noted that the exercises were quite difficult at times and reaching the recommended heart rate zones was challenging. Further, they viewed the group leaders as supportive, motivating, and encouraging and enjoyed the explanation and demonstration of exercises. With respect to peer social support, completers largely felt like they were part of the group but did not get to know their peers (e.g., they found that interactions felt contrived). They thought goal-setting sessions helped with accountability (to work towards goals) and motivation; however, completers were mixed in their view of group-based goal-setting (i.e., some liked suggestions from leaders and peers while others preferred to focus solely on their preferences). Completers enjoyed using the Fitbit devices to track steps. Some felt that they received sufficient instructions on using the devices while others did not understand certain Fitbit features. Completers described either walking outside or doing the exercise videos (provided by group leaders) for their home-based walking sessions depending on weather and convenience.

**Fig. 1:**
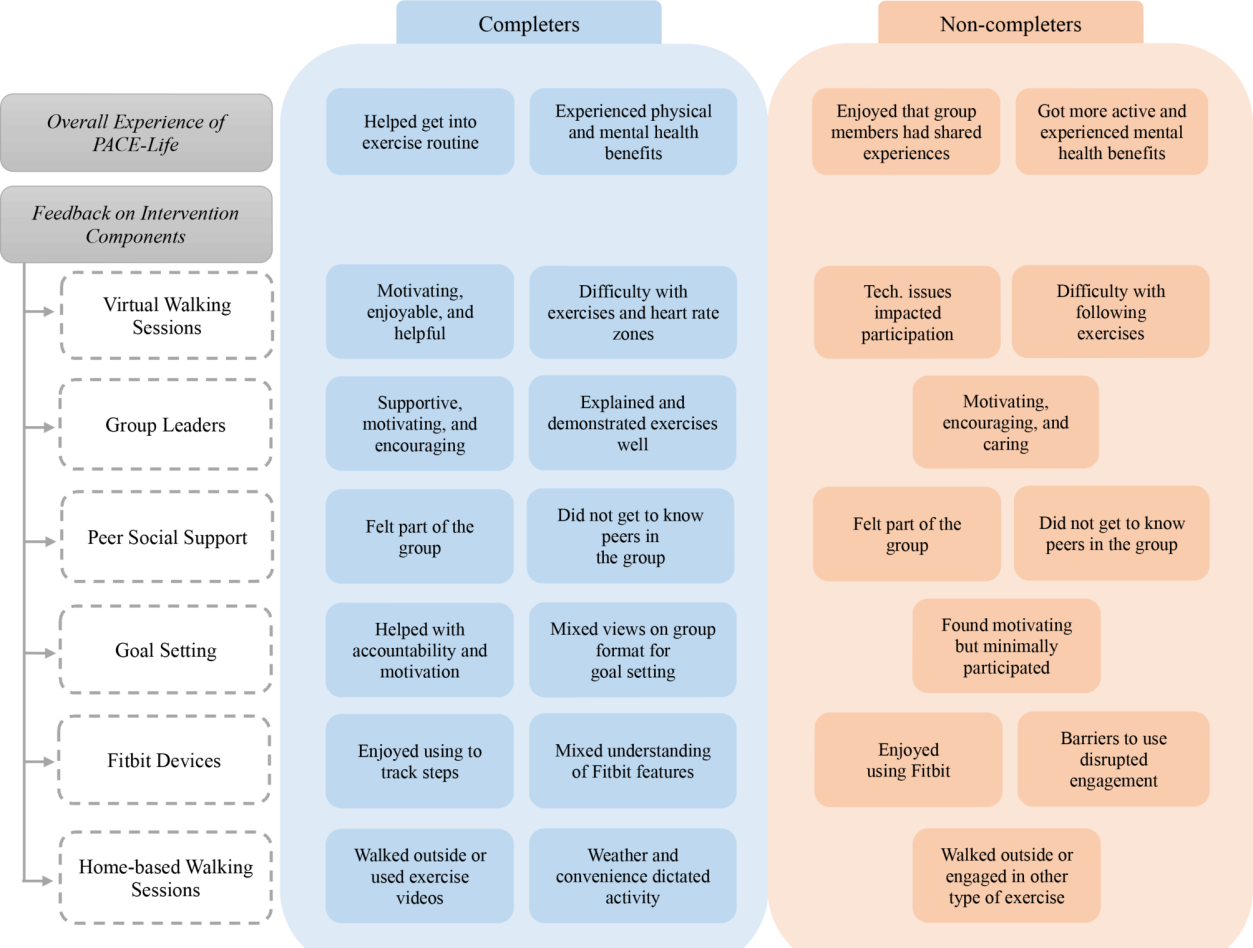
Feedback on the Virtual PACE-Life Intervention by Completer Status

Overall, completers reported that PACE-Life helped them get into an exercise routine and as a result, they experienced physical (e.g., weight-loss) and mental (e.g., reduced anxiety and better mood) health benefits (Fig. 1). One participant shared how the program overall and goal-setting, in particular, helped them modify their routine to include exercise:

“*Because it gave me a reason to exercise… something to do and something to hit. It’s so easy for me to say ‘oh I’ll just throw the dogs in the backyard.’ Where this was like okay, I need to meet my goals. I need to take my dogs to the dog park, you know? So it was nice to have that*.”

#### Non-completers

In terms of the specific components of PACE-Life, non-completers reported that technological issues (e.g., having difficulty getting audio to function properly on tablet) impacted their participation in virtual walking sessions. Further, they found that the exercises were difficult to follow either because they were too complicated or too tiring; however, they reported feeling better after the sessions ended. They viewed the group leaders as motivating, encouraging, and caring. In terms of peer social support, non-completers felt like they were part of the group and liked that group members had shared similar experiences; however, they reported feeling that they did not get to know each other. Non-completers found goal-setting motivating but also described limited participation in the sessions due to virtual session non-attendance. Non-completers enjoyed using the Fitbit devices but expressed barriers to using them regularly (e.g., technological issues, concerns about being tracked). Non-completers described both walking outside and engaging in various other types of exercise (e.g., biking, dancing) during their home-based walking sessions.

Overall, non-completers found PACE-Life enjoyable, valued being in a group with people with similar experiences, and reported becoming more active and noticing improved mental health (Fig. 1). One participant described their overall positive experience with the program:

“*I’ll say that I’m hoping that the study as a whole will benefit the population I’m in with mental health. Like because often times with the medicines we have to take, there’s so much weight gain and just people getting out of shape. And that’s not really addressed typically in traditional treatment. I’ve never been in anything quite like this as far as the focus that it put on it. Like it really had me focused on exercising and that was always kind of an afterthought for me*.”

### Barriers and facilitators to engagement in virtual PACE-life

#### Completers

**Fig. 2:**
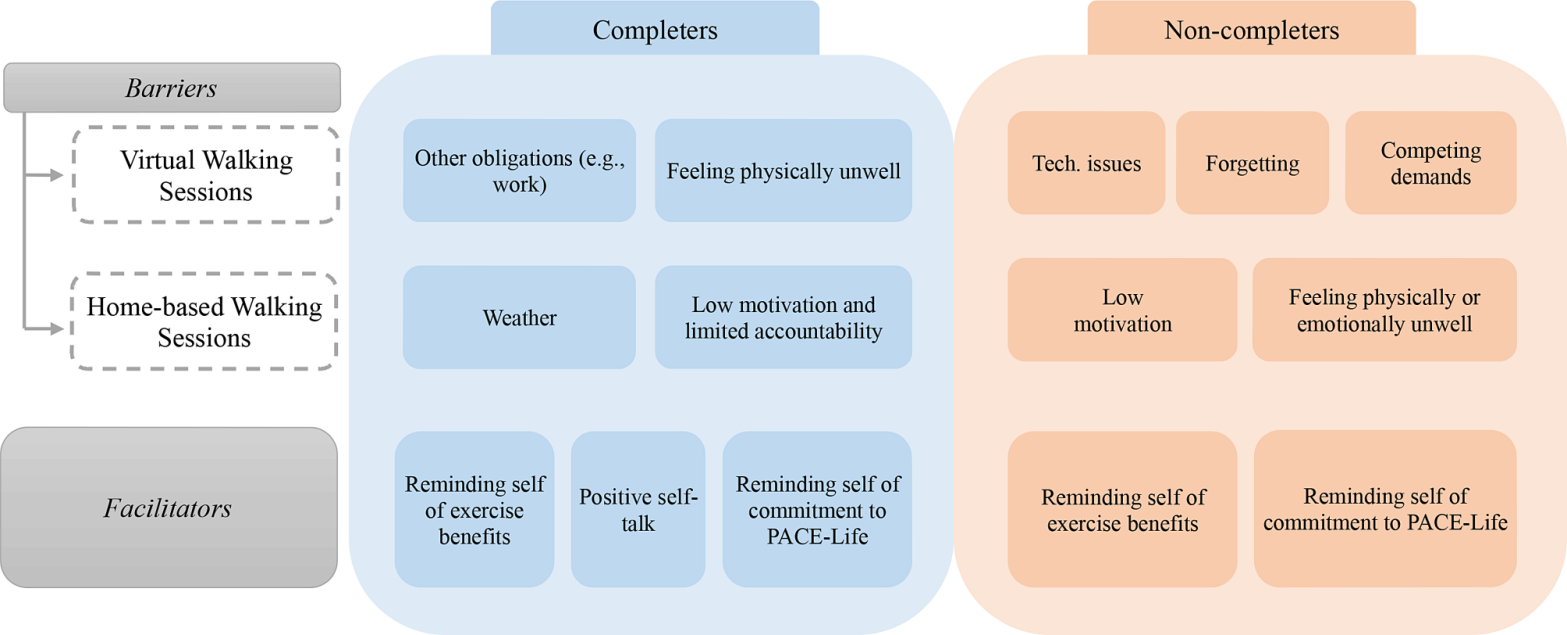
Barriers and Facilitators to Engagement in Virtual PACE-Life by Completer Status

Completers reported that other obligations and competing demands (e.g., work and appointments) as well as feeling physically unwell (e.g., illness, fatigue, pain) impacted their ability to attend virtual walking sessions. For home-based walking sessions, they explained that weather, low motivation, and limited accountability (i.e., to other people/scheduled sessions) were barriers. Completers described using three main strategies to facilitate engagement in walking sessions: (1) reminding themselves of the physical and mental health benefits of exercise, (2) using positive self-talk and encouraging phrases, and (3) reminding themselves of their commitment to the PACE-Life program (Fig. 2). One participant shared examples of the positive self-talk phrases they used to motivate themself to engage:

*“So I just had to tell myself*, *you know*, *you can do it you know. You’re special. Just keep going at it. That’s what I would tell myself. To keep moving.”*

#### Non-completers

Non-completers reported that technological issues, forgetting, and competing demands (e.g., work, time with friends) impacted their attendance at virtual walking sessions. They described low motivation and feeling physically or emotionally unwell as the main barriers to engaging in home-based walking. Two main strategies were noted as facilitators to engagement: (1) reminding themselves of the benefits of exercise, and (2) reminding themselves of their commitment to the PACE-Life program (Fig. 2). One participant highlighted how mental health challenges interfered with engagement:

“*Then there was the factor that I’d feel really down, and I just didn’t want to do anything at one point.*”

### Recommendations for intervention refinement and modality preference

#### Completers

Completers recommended that PACE-Life have greater flexibility in its scheduling of group walking sessions, adding dedicated time for group members to socialize, and including external incentives (e.g., money, group celebrations). Overall, completers reported a preference for virtual exercise programming because it is convenient (i.e., does not require transportation), allows for exercise to occur with comfort and privacy at home, and is a safer option during the pandemic. One participant expressed how the virtual modality allowed for more comfort in one’s own home, especially during the pandemic:

“*Virtual is like the comfort of your area. And with this coronavirus and stuff going on, I think you can be isolated. You can probably even be in quarantine away and still be able to exercise some or I think the virtual as of now might be the way to go*.”

#### Non-completers

Non-completers recommended that the PACE-Life group schedule be more flexible and that the exercise movements be simplified so that they are more doable and easier to follow. Overall, non-completers reported a preference for in-person outdoor exercise programming because it promotes greater social interaction opportunities and allows for more excitement/adventure (e.g., can walk on different paths/trails). One participant explained how in-person exercise would have social benefits:

“*And I think it should be a lot more in person because I think that people will benefit a lot more when you meet your colleagues that live with what we live with. And you get to interact with them one-on-one instead of over a cell phone or tablet*.”

#### Key similarities and differences between groups

In terms of similarities, both completers and non-completers reported a positive experience of Virtual PACE-Life. In particular, they found group leaders and goal-setting sessions motivating, enjoyed using the Fitbit devices, and reported walking outside as their main activity during home-based walking sessions. Both groups found virtual walking sessions difficult and did not feel like they got to know their peers. Motivation was a central barrier to engaging in home-based sessions and reminding themselves of the benefits of exercise and their commitment to PACE-Life were valuable facilitators of engagement. Both groups recommended greater schedule flexibility for future iterations of PACE-Life.

In terms of differences, completers cited other obligations (e.g., work) and feeling unwell as barriers to attending virtual sessions whereas non-complerers noted technological issues, forgetting, and competing demands. Further, in addition to low motivation reported by both groups, completers noted that weather and low accountability (to group leaders/peers/scheduled sessions) impacted their participation in home-based walking sessions whereas non-completers noted feeling unwell as a barrier. Completers reported using positive self-talk as a strategy to increase engagement along with the two additional strategies (reminding self of exercise benefits and commitment to PACE-Life) employed by both groups. Completers recommended that future iterations of PACE-Life include time for socialization and external incentives whereas non-complers suggested that exercises be simplified during virtual sessions. Finally, completers reported a preference for virtual exercise programs and non-completers reported a preference for in-person exercise programs.

## Discussion

This qualitative study explored the perspectives of 15 adults with schizophrenia that participated in a virtual group-based walking program, nine that attended at least 50% of virtual walking sessions (“completers”) and six that attended less than 50% of virtual walking sessions (“non-completers”). Overall, both completers and non-completers viewed their experience in Virtual PACE-Life positively; however, they both found the virtual walking sessions challenging to complete and inadequate for social interaction. Completers and non-completers most notably differed in their reported barriers to engagement and preferences for exercise program modality (i.e., virtual or in-person). These findings have important implications both for future iterations of Virtual PACE-Life and for additional virtual programs designed for those with schizophrenia.

Both completers and non-completers enjoyed their experience in Virtual PACE-Life overall and reported noticing health benefits because of their participation. Both groups especially valued the support, motivation, and accountability from group leaders; however, they did not experience sufficient social support from or interaction with other group members. The positive view of group leaders is encouraging given recommendations for supervised exercise programs designed for this population [10] and previous reports that staff support is helpful for facilitating physical activity participation in this population [16, 32]. Yet, the lack of peer social support is disappointing given that opportunities for social interaction have been found to be a key reason many people with schizophrenia engage in exercise [17]. Unlike in-person walking groups, the virtual group environment limits opportunities for conversation during exercise (verbally or using the chat function) because participants are focused on following the group leaders’ physical activity instructions. Further, as virtual platforms allow one speaker to be heard at a time, multiple conversations within the group are unlikely to occur. Similar challenges of facilitating social interactions in virtual groups have been reported in psychotherapy for people with schiozphrenia [33], further highlighting the need for strategies to enhance the social aspect of virtual group-based treatments for this population.

Both completers and non-completers found the virtual walking sessions especially challenging to complete either because they were too physically difficult or because the movements were too complicated. These findings may help to explain the lack of improvement in cardiorespiratory fitness observed in the pilot RCT in that participants likely did not achieve and/or maintain an adequate exercise intensity during groups [22] because they were too challenging. These results were unexpected given that PACE-Life walking sessions gradually increased in duration and exercise intensity over the course of the intervention and group leaders offered modifications during sessions to simplify movements. Physical activity levels substantially declined during the pandemic [34], which may have contributed to our participants’ difficulty completing the walking-based movements. It is possible, though, that the virtual delivery of walking-based movements made it harder for participants to follow along than would be the case in an in-person program. As such, future iterations of Virtual PACE-Life should consider the baseline exercise levels of participants and should consistently review movements (allowing time for participants to ask questions) to optimize their physical activity engagement.

Completers and non-completers both noted that low motivation impacted their ability to complete home-based walking sessions; however, they diverged on barriers to attending virtual walking sessions. Completers found other obligations (e.g., work) to be the main obstacle to attendance whereas non-completers noted technological issues and forgetting as well as competing demands. Executive functioning, which involves planning and working memory among other processes [35], may explain differences in the barriers noted between the two groups. In fact, non-completers had lower education levels and lower rates of employment, characteristics that are also associated with reduced executive functioning in schizophrenia [36]. As such, including an assessment of executive function in future virtual exercise trials in persons with schizophrenia may be valuable to help identify participants that may benefit from additional supports like ongoing technological support and reminders. Further, given that executive functioning is reduced in schizophrenia [37] and exercise has been shown to improve multiple aspects of cognition in this population [3], enhancing engagement for those with lower cognitive abilities may be especially beneficial.

Both groups recommended that the schedule of group sessions have greater flexibility to facilitate better engagement. Completers also suggested adding dedicated time during groups for socialization and including external incentives to increase motivation whereas non-completers suggested simplifying the exercise movements so that they were easier to complete. Further, with respect to modality preferences for future physical activity programs, completers wanted virtual exercise programming whereas non-completers wanted in-person (rather than virtual) programming. Overall, these recommendations in tandem with reported barriers to participation suggest the need for greater choice and stronger peer support in physical activity interventions designed for this population.

Future research should consider more options for when and how individuals participate in walking sessions. Specifically, it could be helpful to include drop-in sessions, which have been recommended in previous work [32] as these would enhance accessibility of sessions and accomodate participants’ competing demands. Additionally, given the differing preferences for virtual versus in-person modalities, allowing participants to select the delivery method for walking sessions may increase engagement. As suggested by participants, allowing adequate opportunities for conversation prior to the start of the walking session that focuses on building relationships may be especially important so that individuals are able to learn about each other and gain comfort within the group. Further, a non-randomized feasibility study of virtual group exercise for people with schizophrenia allowed participants to have a guest (e.g., friend or family) join them for the virtual pilates or fitness sessions [38], which may aid in providing greater social support through the presence of familiar relationships. These possible changes not only respond to the recommendations offered by participants in our study but they also have the potential to enhance autonomy (i.e., by providing greater choice) and relatedness (i.e., by strengthening the social environment), two core aspects of self-determination theory, the theory of motivation that guided the design of Virtual PACE-Life.

The study had three main limitations. First, this work was conducted during the COVID-19 pandemic, which likely impacted participants’ experience of and engagement in the exercise program. Second, our sample size of 15 is relatively small, particularly when split into completers (*n* = 9) and non-completers (*n* = 6). Third, although reading level was assessed for inclusion in the study, specific measures of cognitive ability (e.g., executive functioning) were not included. Despite these limitations, this qualitative study offers valuable information on virtual exercise programming for adults with schizophrenia that can inform intervention development efforts for this population. Future research should consider modifying the Virtual PACE-Life program to improve the social aspect and include more choices with respect to how and when sessions are completed. Future studies should also consider using baseline cognition and exercise testing to further guide appropriate person-specific modifications to the program.

## Data Availability

The datasets used and/or analysed during the current study are available from the corresponding author on reasonable request.

## Abbreviations

PACE-Life: Physical Activity Can Enhance Life
RCT: Randomized controlled trial
